# DNA bank for Polish patients with predispositions to occurrence of colorectal polyposis

**DOI:** 10.1186/1897-4287-9-S2-A8

**Published:** 2011-06-01

**Authors:** A Pławski, M Podralska, R Słomski

**Affiliations:** 1 Institute of Human Genetics, Polish Academy of Sciences, Poznan, Poland

## 

Intestinal polyposis syndromes comprise of a group of diseases conditioned by the occurrence of hereditary mutations. In the Polish DNA bank of hereditary predisposition to polyposis we collected DNA samples derived from persons from families with a diagnosed adenomatous polyposis including familial adenomatous polyposis coli together with its recessive form, Turcot’s syndrome, inherited mixed polyposis as well as persons with recognised hamartomatous polyposis containing juvenile polyposis, Peutz-Jeghers syndrome, Cowden syndrome and Proteus syndrome. Investigations were conducted on DNA isolated from the peripheral blood cells. The search for mutations in *APC*, *MUTYH*, *PTEN*, *BMPR1A*, *SMAD4* and *STK11* genes preconditioning the occurrence of individual diseases was performed employing PCR-SSCP, PCR-HD, DHPLC as well as RFLP techniques and DNA sequencing. At the present time, the DNA Bank comprises the total of 1097 DNA samples derived from 449 families with intestinal polyposis of which 945 samples come from persons in whose families Familial Adenomatous Polyposis (FAP) occurred. In addition, the collected data also contain material derived from 25 families with Peutz-Jeghers syndrome and from 20 families with juvenile polyposis as well as single cases with the Cowden syndrome, Proteus syndrome and desmoid tumors. The performed molecular investigations allowed identification of mutations from 44 to 50% studied families. With regard to the quantity of the material collected for analyses and the efficacy level of the employed molecular methods, the obtained results are in keeping with the results found in the literature from the field of genetics and medicine and do not differ from world standards. The collection of data and materials for investigations in the case of rare diseases allows qualitative, organisational and economic optimisation of the performed investigations.

